# Moving towards social inclusion: Engaging rural voices in priority setting for health

**DOI:** 10.1111/hex.13895

**Published:** 2023-10-26

**Authors:** Aviva Tugendhaft, Nicola Christofides, Nicholas Stacey, Kathleen Kahn, Agnes Erzse, Marion Danis, Marthe Gold, Karen Hofman

**Affiliations:** ^1^ SAMRC/Wits Centre for Health Economics and Decision Science—PRICELESS SA School of Public Health, Faculty of Health Sciences, University of Witwatersrand Johannesburg South Africa; ^2^ School of Public Health Faculty of Health Sciences, University of the Witwatersrand Johannesburg South Africa; ^3^ Department of Health Policy London School of Economics London UK; ^4^ MRC/Wits Rural Public Health and Health Transitions Research Unit—Agincourt School of Public Health, Faculty of Health Sciences, University of the Witwatersrand Johannesburg South Africa; ^5^ Department of Bioethics National Institutes of Health Bethesda Maryland USA; ^6^ New York Academy of Medicine New York City New York USA

**Keywords:** priority‐setting, public engagement, rural health, social inclusion, South Africa

## Abstract

**Background:**

Achieving universal health coverage (UHC) in the context of limited resources will require prioritising the most vulnerable and ensuring health policies and services are responsive to their needs. One way of addressing this is through the engagement of marginalised voices in the priority setting process. Public engagement approaches that enable group level deliberation as well as individual level preference capturing might be valuable in this regard, but there are limited examples of their practical application, and gaps in understanding their outcomes, especially with rural populations.

**Objective:**

To address this gap, we implemented a modified priority setting tool (Choosing All Together—CHAT) that enables individuals and groups to make trade‐offs to demonstrate the type of health services packages that may be acceptable to a rural population. The paper presents the findings from the individual choices as compared to the group choices, as well as the differences among the individual choices using this tool.

**Methods:**

Participants worked in groups and as individuals to allocate stickers representing the available budget to different health topics and interventions using the CHAT tool. The allocations were recorded at each stage of the study. We calculated the median and interquartile range across study participants for the topic totals. To examine differences in individual choices, we performed Wilcoxon rank sum tests.

**Results:**

The results show that individual interests were mostly aligned with societal ones, and there were no statistically significant differences between the individual and group choices. However, there were some statistically significant differences between individual priorities based on demographic characteristics like age.

**Discussion:**

The study demonstrates that giving individuals greater control and agency in designing health services packages can increase their participation in the priority setting process, align individual and community priorities, and potentially enhance the legitimacy and acceptability of priority setting. Methods that enable group level deliberation and individual level priority setting may be necessary to reconcile plurality. The paper also highlights the importance of capturing the details of public engagement processes and transparently reporting on these details to ensure valuable outcomes.

**Public Contribution:**

The facilitator of the CHAT groups was a member from the community and underwent training from the research team. The fieldworkers were also from the community and were trained and paid to capture the data. The participants were all members of the rural community‐ the study represents their priorities.

## 1 INTRODUCTION

The 2030 Agenda for Sustainable Development is driven by the global commitment to ‘leave no one behind’ and ‘put the furthest behind first’.^1^ This agenda includes promoting social inclusion and prioritising the poorest and most disadvantaged groups in society.^2^ The sustainable development goals (SDGs) in turn were developed to address inequities and SDG 3 aims to ‘ensure healthy lives and promote well‐being for all’.^1^ As such, there has been a global commitment towards Universal Health Coverage (UHC) to ensure everyone has access to quality healthcare without financial hardship. This is more pertinent in rural areas where 80% of people living in poverty reside and health inequities prevail.^3^ It is well established that people in the lowest socioeconomic groups, many of which reside in rural areas, are more vulnerable to disease and illness (‘illness vulnerability’) and experience greater lack of access to health care (‘access vulnerability’), especially in low‐ and middle‐income settings thereby leading to greater social exclusion.^4^, ^5^, ^6^

While social exclusion is a contested term, it broadly refers to a state where individuals are unable to participate fully in society due to lack of access to material resources (income employment, housing) and services such as healthcare.^5^ It also extends to a lack of voice in decision making that impacts their lives.^5^ Rural populations often experience greater social exclusion due to these factors.^7^ Social inclusion, on the other hand, refers to the process of improving the terms for individuals and groups to participate in society through improved access to resources and services, opportunities as well as voice and respect for rights.^5^

Achieving UHC in the context of limited resources requires addressing inequities and fostering social inclusion through the design and implementation of health services packages that prioritise the most vulnerable, including rural populations.

South Africa is on the path to UHC to be funded through a National Health Insurance (NHI) scheme. The NHI Bill declares that priority should be given to groups that are in greatest need and experience greatest difficulty in obtaining care.^8^, ^9^ This will require prioritising vulnerable groups including rural populations. In the country, the overall disease burden is higher for those living in poverty, 75% of which reside in rural areas, largely due to entrenched structural inequalities and is exacerbated by poor access to services and greater social exclusion.^6^, ^10^ These healthcare access challenges are also often greater in rural areas compared to urban areas.^11^ While there is an overriding commitment to prioritise the most vulnerable there is at times a disconnect between what decision makers believe is most important to these groups and what their lived reality is.^12^ The success of NHI in the context of limited resources will depend on appropriate, justifiable, and acceptable decisions to ensure that those in greatest need are reached, simultaneously with public buy‐in. One way to ensure identified interventions respond to the needs and values of the population and to increase the acceptance and success rate of these interventions is to give individuals, especially within vulnerable populations, greater control, and agency in designing health services packages. Doing so would enable individuals to participate in the priority setting process where they are able to appreciate the implications of budget constraints and inevitable trade‐offs.

Globally, there is increasing recognition that public values ought to be considered in a priority setting for health^13^, ^14^, ^15^ with different approaches proposed for eliciting these values and prioritising related interventions. These approaches focus either on individual level preferences that are aggregated, or on group level deliberation to reach consensus or a final solution.^16^ Both approaches are intended to overcome the challenge of competing perspectives in determining public preferences but also have their limitations. Aggregation fails to consider the reasons and justifications behind the preferences and makes no provision for shifting of priorities after considering others' views. Deliberation, while resulting in priorities that are based on informed and reasoned exchange and debate between individuals, may not always succeed at ensuring true representation and may overlook some important preferences especially if vulnerable groups are not adequately represented.^13^, ^16^ Deliberation also at times fails to consider dissenting views which may exist even after a group solution is reached, and may be overshadowed by stronger voices, or undermined by social pressure and confirmation biases.^17^, ^18^ Some have called for an approach that combines both these elements and aims to reach a broader public by considering individual level preferences as well as group decisions.^16^ Aggregated individual level priority setting may demonstrate alignment as well as differences with group choices, and individual characteristics like gender, level of education, age, income level and health status may be important to understand what drives some of these decisions. Where plurality exists, which is often inevitable with public engagement in priority setting, it may not always be possible to reconcile differences. However, legitimacy and acceptability of the decision‐making process can be enhanced through ensuring transparency which would involve detailed reporting on the process including participant characteristics, as well as where conflicts emerged and how, or if they were resolved.^17^

This paper aims to explore the outcome of a public engagement process for priority setting for health using a tool called CHAT (Choosing All Together) in terms of the choices and priorities of individuals as compared to group choices, as well as the sociodemographic characteristics that influenced decisions at the individual level. The paper considers how priorities might differ amongst individuals even within the same rural community, and the importance of transparency in this regard. This is the first time such a tool has been implemented in a rural community in South Africa.

Some previous analyses from the implementation of the CHAT tool have compared individual and group choices and two analyses considered the sociodemographic characteristics associated with levels of investment for priorities.^19^, ^20^, ^21^, ^22^ This paper adds to the body of work by considering the individual level health preferences of a rural community and demonstrates that even within this community where individual alignment with group priorities is potentially high, different preferences may exist among individuals with different sociodemographic characteristics. The paper provides insight into how marginalised voices, as well as broader publics, may be meaningfully incorporated into the priority setting agenda.

## 2 METHODS

### 2.1 Study site

The Agincourt Health and Socio‐Demographic Surveillance System (HDSS) study area (https://www.agincourt.co.za/), platform of the MRC/Wits Rural Public Health and Health Transitions Research Unit since 1992, is in Bushbuckridge sub‐district in Mpumalanga Province. The area is typical of rural South Africa, characterised by poverty and underdevelopment as well as a strong traditional authority.^23^ It has a population of approximately 116,000 residing in 31 villages where life expectancy at birth is 68 for males and 74 for females.^24^ Infant mortality rate is 39.1 and under 5 mortality rate is 10 per 1000.^25^ The area is characterised by a quadruple burden of disease of HIV/AIDS and tuberculosis (TB); noncommunicable diseases (NCDs); maternal, perinatal, and nutritional conditions; and injuries. There are two health centres, six satellite clinics, and three district hospitals within 20–60 km from the villages. Sanitation systems are inadequate with 53% of the 20,000 households receiving pipe‐borne water.^26^ Tarred roads now link many of the villages but are maintained poorly and gravel roads are present within the villages. Every village has at least one primary school and most have a high school, but the quality of education is poor^22^ with 54.9% of adults having passed matric. Unemployment rates are high, and many households are dependent on government welfare grants.^26^

### 2.2 Materials

We used a modified priority setting tool called Choosing All Together (CHAT) SA that enables individuals and groups to make trade‐offs using a limited budget to demonstrate the type of health services packages that may be acceptable to a rural population.^27^, ^28^ The tool was originally developed by the US National Institutes for Health and Michigan University as a priority‐setting simulation exercise.^29^ During the simulation, a trained facilitator guides participants through different rounds (individual rounds and a group round) where they distribute a limited number of stickers on a board as they select from a range of options. The stickers, which represent the available budget, are only able to cover approximately 60% of the options on the board, the cost of which is represented by holes on the board.

The tool was modified for use in Bushbuckridge and described elsewhere.^27^ In brief, this modification process included an iterative participatory approach that relied on policy analysis and engagement with experts and community members to identify health topics and related interventions specific to the Bushbuckridge context, as well as a costing component. The outcome of the modification process was a context specific, bilingual CHAT board that included seven health topics/issues and related interventions within each topic/issue to select from as part of a health services package through the allocation of funds represented by stickers.

The CHAT SA board is included in Supporting Information: Appendix A and the health package options are summarised in Supporting Information: Appendix B. The board is divided into pie slices with different icons for each slice. Each pie slice represents a health topic or issue and are further divided according to different categories of interventions represented by numbers: health promotion (1), prevention (2), diagnosis (screening) (2), treatment (3), rehabilitation (3), and palliative care (4).^30^ The access slice included five unique categories (numbered one to five on the board) (see Supporting Information: Appendices A and B for specific interventions and categories). The total cost of the package of interventions is approximately R2 billion ($123 million) represented by 67 holes and each category of interventions per health topic/issue has a specific cost depicted by the sticker holes. Participants received 35 stickers that represented the funds they had available to allocate and that were able to cover 52% of the options on the board. This allocation was based on a starting point of 60% of stickers drawing on past CHAT exercises and was revised to allow for more meaningful rationing in the context of this specific board. The categories and specific interventions for each category were explained in detail in a user manual for each participant written in simple language and in the local language.

### 2.3 Sampling

Purposive sampling was used to ensure a range of age groups from different villages with different levels of infrastructure development and barriers to healthcare access, as well as a mix of men and women. The villages included three with clinics and three without; three with tarred roads and three with dirt roads. The sampling was conducted in this way to encourage diverse perspectives from this rural community.

Sixty‐three individuals participated in seven group deliberations using CHAT, with 6–11 individuals in each group. There was a mix of women and men in each of the groups except for two which included one group with only older (55 years) men and another group which included predominantly younger (23 years) women. There was another older group (52 years) with men and women, a younger group (25 years) with men and women and two mixed middle age (42 years; 43 years) groups. Table 1 shows the group composition in terms of age and gender of the seven groups.

**Table 1:** Group composition in terms of gender and age.

	G 1	G 2	G 3	G 4	G 5	G 6	G 7
Male	1	3	3	1	7	5	7
Female	5	5	8	10	4	4	0
Age range (years)	37–62	30–67	30–55	20–28	20–42	40–66	48–67
Mean age (years)	42	43	39	23	25	52	55

### 2.4 Study procedures

Before the CHAT exercise all participants in each group completed a short self‐administered demographic questionnaire. The CHAT tool was used over three rounds. During round 1, after the facilitator explained the board and the accompanying user manual, participants individually allocated 35 stickers to the health issues and interventions that they perceived to be the highest priorities for their own family. Once this was complete, the group completed a board collectively (with 35 stickers) in terms of their priorities for the entire community of Bushbuckridge (round 2: group round). Scenario cards were used by the facilitator to assist participants in thinking through the implications of the decisions that they made. During this group round, guided by the facilitator, participants discussed the topics in depth and deliberated with one another to finally reach agreement (by majority vote) for the group allocations. Qualitative data was captured during the group rounds and analysed as part of a separate paper.^28^ During the final round (round 3), participants were again asked to complete the exercise individually (using 35 stickers) thinking about which priorities they believed were most important for their own family. At the end of the exercise the facilitator asked some brief feedback questions to the group before concluding. The entire exercise took half a day to complete. Supporting Information: Appendix C summarises the various steps of the CHAT process.

### 2.5 Statistical analysis

We used descriptive statistics to describe the study participants using the data from the questionnaire. The sticker allocations of all study participants were recorded at each stage of the study. From these the number of stickers allocated to each topic by the participants was calculated by adding up the number of stickers across interventions selected by the participant for the particular topic. For the group round, the value for each respondent was the value of their group. We calculated the median and interquartile range across study participants for the topic totals. The median referred to the median across all respondents for the particular round where *n* = 63. To examine differences in sticker allocations, we performed Wilcoxon's rank sum tests for differences across participant categories and sticker allocations in round 3. The participant categories were specified as priori based on the demographic data from the questionnaire. Results are reported to be statistically significant if *p* < .05. All statistical analyses were conducted using STATA SE v 15.1.

## 3 RESULTS

### 3.1 Participants

Table 2 shows the participants' characteristics. Participants (*n* = 63) ranged in age between 20 and 69 years with a mean age of 39 years, and there were more women (57%) than men (43%). Twenty‐seven percent had a primary school level or no schooling and 73% had high school or above. Most households (57%) earned R3000 ($170) or below per month and were dependent on either solely government grants or a combination of grants and employment.

**Table 2:** Participant characteristics.

Participant characteristics	*n*	%
Age		
20–45	39	62
46–69	24	38
Gender		
M	27	43
W	36	57
Education		
No school	7	11
Primary school	10	16
High school	41	65
Tertiary	5	8
Household income		
R3000 and below	36	57
R3001–R5000	17	27
>R5000	10	16
Income source		
Government grants	19	30
Employment	11	17
Grants and employment	23	37
Other	10	16

### 3.2 Individual and group investments

#### 3.2.1 Differences and similarities across rounds

Allocation of stickers by topic across all three rounds were very similar (Table 3). Individuals in round 1 allocated a median of nine stickers to NCDs, with more of a range, which increased to 17 in the group round and remained the same at 17 in the final individual round with a similar range. For HIV/AIDS and TB the median sticker allocation was 12 in all rounds, but the range was higher in the individual rounds. Sticker allocations for other topics remained even more similar across rounds.

**Table 3:** Number of stickers allocated by topic across rounds.

	Round 1	Round 2	Round 3	
	Median (IQR)	Median (IQR)	Median (IQR)	*p* Value
MNRH3	3 (2–5)	2 (1–3)	3 (2–4)	.041
Child health	2 (1–3)	1 (1–3)	2 (1–3)	.88
HIV/AIDS and TB	12 (11–16)	12 (11–12)	12 (6–12)	.32
NCDs	9 (6–17)	17 (8–17)	17 (7–17)	.21
Access	4 (1–7)	5 (1–6)	4 (2–5)	.63
Abuse	1.5 (1–2)	2 (1–2)	2 (1–2)	.76
Malaria	2 (1–3)	2 (1–3)	2 (1–3)	.54

Abbreviations: NCD, noncommunicable disease; TB, tuberculosis.

### 3.3 Participant characteristics and patterns in investment levels

Among the participant characteristics—which included age, gender, education level, and income level—age was significantly associated with levels of investment in the different health issues during the final individual round. The difference across age groups is statistically significant for MNRH, Child health, Access, and Malaria. Older age groups were more inclined to invest in these topics as well as for Abuse as indicated by a higher number of stickers allocated. For HIV/AIDS and NCDs the investment through the sticker values decreased with older age groups (Table 4).

**Table 4:** Participant characteristics and number of stickers allocated by topic, round 3.

	Age **≥**45	Age <45		Men	Women		Income Above R3000	Income R3000 and below		High school and above	Primary school and below	
	Median	Median		Median	Median		Median	Median		Median	Median	
	(IQR)	(IQR)	*p* Value	(IQR)	(IQR)	*p* Value	(IQR)	(IQR)	*p* Value	(IQR)	(IQR)	*p* Value
MNRH	4.00	2.00	.003	3	3	.33	3.00	3.00	.39	3.00	4.00	.083
	(2.00, 5.00)	(1.00, 3.00)		(2–4)	(1–3.5)		(2.00, 3.00)	(1.50, 4.50)		(2.00, 3.00)	(2.00, 5.00)	
Child health	2.00	1.00	.008	1	2	.1	1.00	2.00	.40	1.00	2.00	.10
	(1.00, 3.00)	(1.00, 2.00)		(1–3)	(1–3)		(1.00, 2.00)	(1.00, 3.00)		(1.00, 3.00)	(1.00, 3.00)	
HIV/AIDS and TB	11.00	12.00	.47	12	12	.82	11.00	12.00	.019	12.00	11.00	.91
	(5.00, 12.00)	(11.00, 12.00)		(6–12)	(11–12)		(5.00, 12.00)	(8.00, 15.00)		(5.00, 12.00)	(5.00, 15.00)	
NCDs	13.00	17.00	.38	17	17	.72	17.00	13.00	.033	17.00	9.00	.45
	(7.00, 17.00)	(8.00, 17.00)		(7–17)	(8–17)		(17.00, 17.00)	(7.00, 17.00)		(8.00, 17.00)	(6.00, 18.00)	
Access	4.00	3.00	.41	3	4	.92	3.00	4.00	.52	3.00	4.00	.18
	(2.00, 5.00)	(2.00, 5.00)		(2–5)	(2–5)		(2.00, 5.00)	(2.00, 5.00)		(1.00, 5.00)	(3.00, 5.00)	
Abuse	2.00	2.00	.75	2	2	.67	2.00	1.50	.90	2.00	2.00	.89
	(1.00, 2.00)	(1.00, 2.00)		(1–2)	(1–2.5)		(1.00, 2.00)	(1.00, 2.00)		(1.00, 2.00)	(1.00, 2.00)	
Malaria	2.00	1.00	.059	2	2	.48	2.00	2.00	.45	1.00	2.00	.024
	(1.00, 3.00)	(1.00, 2.00)		(1–3)	(1–2.5)		(1.00, 3.00)	(1.00, 3.00)		(1.00, 2.00)	(2.00, 3.00)	

Individual income was statistically significantly associated with the investment in health issues as indicated by the sticker allocations for NCDs and HIV/AIDS. Those earning R3000 and below invested less in NCDs and more in HIV/AIDS than those earning above R3000. Gender was not significantly associated with any of the allocations made and education was only statistically significant for Malaria (Table 4).

## 4 DISCUSSION

The implementation of the CHAT SA tool in a rural community shows that individual interests were aligned in many ways with societal ones as indicated by the similarities across the individual and group rounds with no statistically significant differences. Yet there were some differences within the individual rounds, which could be based on individual characteristics. In particular, older age groups invested more in MNRH, Child Health, Access, Malaria while younger age groups invested more in NCDs and HIV/AIDS. Those with higher levels of education invested more in HIV/AIDS & TB and NCDs and those with lower incomes invested more in HIV/AIDS and TB and less in NCDs.

The similarities between the group and individual rounds could be explained by high levels of social integration at the community level,^31^ which may be like other rural settings. Some previous CHAT analyses which compared individual and group rounds showed that individuals changed their preferences in the final round to be more aligned with the group choices.^32^ while others demonstrated that individuals reverted to their initial preferences even though these differed from the group.^22^, ^33^ These studies were conducted in high income settings which differ considerably to low‐income rural settings where community‐level social cohesion may not be as high. Our study adds to the body of work and demonstrates the importance of capturing the details of public engagement processes and transparently reporting on these details for the outcomes to be valuable.

Within the final individual rounds there are some differences in levels of investment among participants. Younger participants invested more in NCDs and HIV/AIDS and TB and less in MNRH, Child health, Access and Malaria as compared to older participants. This demonstrates that individuals were at times not only driven by self‐interest and were considering broader, perhaps societal implications, of their choices. A further reason might be because this community includes multigenerational households^34^ and strong family ties which results in younger family members providing care for older family members, as well as grandmothers providing primary caregiver roles to grandchildren. Therefore, investments in interventions targeted at different age groups would be beneficial to the family unit (or appreciated by those even without a direct benefit).

In terms of Access, older individuals may have invested more in this area due to actual challenges that are experienced such as access to chronic medications as well as travelling long distances to hospitals, which can be even more challenging for the elderly. This indicates that priorities of individuals within vulnerable groups do differ and speaks to the need to ensure diverse age groups are included in priority setting processes.

The CHAT tool is unique in that it allows for both individual level and group level decision‐making. Although choices were similar across rounds in this community, other CHAT exercises, mostly in higher income settings, have demonstrated more divergence. At times, group deliberations have not only impacted group choices but also final individual choices.^20^, ^32^ This demonstrates that individual level and group level processes can influence one another and public engagement in priority setting may be more meaningful as part of an iterative process at various stages of decision‐making.

Our results support other viewpoints that if public engagement in priority setting is viewed as important, the challenges of reconciling plurality will need to be overcome, even within populations with strong levels of social cohesion and alignment.^16^ Group deliberative approaches help to reach consensus and reconcile some of the differences, but additional individual voices may also need to be considered alongside group processes, especially among the most vulnerable. This speaks to the need to ensure representation of the different ‘publics’ in priority setting for health, with strong consideration of the heterogeneity of the South African population, and especially vulnerable populations. Deliberative methods, while successful at capturing reasons behind choices, and developing a deeper understanding of the values of communities, may overlook some important priorities and may not effectively reach enough of the public. Consideration should be given to implementing these tools alongside other individual level engagement mechanisms. In doing so, some of the standard approaches of individual level preference capturing may need to be modified to ensure voices of the most vulnerable, including rural populations, are captured. National level surveys, for example, often rely on the use of devices that are not always appropriate for rural communities who are difficult to reach. These individual‐level engagement mechanisms would also benefit from depicting choices within a constrained budget, otherwise it proves difficult to translate public priorities into policymaking for health.^22^ The outcome of these broader engagements would not be sufficient in determining a final decision but could be useful in guiding decision makers as one component in a broader priority setting process that also considers wider ethical considerations and economic evidence.

Another way to respond to plurality, especially in the context of UHC is to consider multiple health services package options. Others have demonstrated that individuals can live within constraints but in different ways and may support the view for a more individualised approach to UHC where different preferences are considered.^35^ Our analysis shows that age may be one criterion that should be considered in the design of health services packages and different packages may be appropriate for different age groups. More broadly, specific packages for vulnerable populations, including rural groups, might be valuable. Equity considerations are already promoted in priority setting processes and the voice of the vulnerable could be included for this to be more impactful. In South Africa, the national level priority setting for health policies could incorporate vulnerable voices, but at provincial and local level where service delivery decisions are being made rural voices should influence these decisions to ensure they are more appropriate for and responsive to different local settings. Further refining these packages according to additional characteristics like age could be beneficial.

Finally, while plurality may not always be reconcilable, transparently capturing and reporting on public engagement processes can contribute to a deeper understanding of community priorities and competing viewpoints, as well as greater acceptance of final decisions.

This paper demonstrates one approach for potentially improving social inclusion and shows how vulnerable individuals and groups can have their voice included in decision making for healthcare. Incorporating this voice would in itself improve social cohesion, and if the outcome of such exercises were translated into policy and service level decisions then barriers to accessing healthcare would be addressed thereby further promoting social inclusion. Social inclusion, however, is multifaceted and can only be fully addressed by paying attention to the broader political, economic, and social environment. This paper is potentially one step in the right direction, providing lessons on how social inclusion can be improved for vulnerable communities through greater agency and control in health service package design, and in turn can impact illness and access vulnerability.

### 4.1 Limitations

The demographic information we captured was limited and our results could have been strengthened if it had been more extensive. Health status as well as household composition would have been especially valuable as this could have potentially added to our understanding of some of the investment decisions.

Our sample size was small, and it is possible that there was insufficient power to detect small differences between rounds of the deliberative processes. Our sample was not large enough to run multivariable regression models and as a result we were not able to adjust the results for other factors. For example, individuals who were older AND female may have made different choices to older males. In addition, the sample was purposive to ensure representation from different villages on the study site, different age groups and both male and female participants. This could have resulted in selection bias.

A further limitation is that the data points in round 3 are not independent and we did not adjust for correlated data. However, the comparison across rounds demonstrated that the difference between round 1, where the data points are independent, and round 3 was not statistically significant so adjusting for intergroup comparison may not have been necessary.

Group dynamics was not considered in terms of impact on individual level decisions, this should be explored in the future.

## 5 CONCLUSION

Successfully Achieving NHI in South Africa, and making progress towards the 2030 SDG targets, will rely on reaching the most vulnerable by ensuring policies and related interventions are responsive and appropriate and address social exclusion. Public engagement provides an opportunity to ensure these voices are included in the decision‐making processes. Group level and individual level engagement approaches have strengths and limitations, and both may be necessary to ensure accurate capturing of priorities, and underlying values of marginalised groups. The CHAT process demonstrates that rural groups and individuals can grapple with the idea of limited resources and difficult allocative decisions and provides an example of how excluded voices may be meaningfully incorporated in the priority setting agenda. The study shows that even in communities with high levels of social integration, individual level preferences can differ, and these preferences are shaped by different characteristics. This CHAT implementation demonstrates meaningful outcomes that could be useful to policymakers. However, to bolster its utility further, research should focus on extending its implementation with more individuals and groups, within the same rural area as well as beyond, and within urban areas. This could be conducted alongside other aggregation methods. In doing so, a deeper understanding of social values as well as associations between individual preferences and characteristics would emerge which could inform health service package design, especially if policymaker engagement is included from the outset. In addition, efforts around the institutionalisation of priority setting in South Africa would benefit from the equal input of experts in methods of public engagement alongside other technical experts in priority setting. This could lead to more effective decision making, which in turn could improve health outcomes.

## AUTHOR CONTRIBUTIONS

Aviva Tugendhaft, Nicola Christofides, Karen Hofman, Kathleen Kahn, Marion Danis and Marthe Gold conceptualised the study. Aviva Tugendhaft developed data collection tools and materials, performed the analysis; and wrote the paper. Nicola Christofides and Nicholas Stacey provided technical support for data analysis. Agnes Erzse assisted with data collection and material development. Marion Danis provided technical guidance for implementation of the CHAT tool. All authors reviewed the paper.

## CONFLICT OF INTEREST STATEMENT

The authors declare no conflict of interest.

## ETHICS STATEMENT

Ethical approval for the study was obtained from the Human Research Ethics Committee (Medical) of the University of the Witwatersrand, Johannesburg, South Africa [Clearance certificate number M161009]. An informed consent process was undertaken at the recruitment stage. Separate consent was obtained for audio recording. To ensure anonymity, participants were given a participant number that was used throughout the study.

## Supporting information

Supporting information.

Supporting information.

Supporting information.

## Data Availability

Data will be made available upon request
