# Supplementary material for: Moving towards social inclusion: Engaging rural voices in priority setting for health
Source: Health Expect. 2023 Oct 26;27(1):e13895. doi: 10.1111/hex.13895 (PMC10726206; doi:10.1111/hex.13895)
Supplement: Supplementary file 1 — Supporting information. [file HEX-27-e13895-s002.docx]

**Appendix B**: Table of topics/issues, specific interventions and number of holes^*^ of the CHAT board

| **Mother, newborn and reproductive health (MNRH)** | **Number of holes** |
| --- | --- |
| ***1: Education and information*** | **1** |
| Two-month long media campaign on antenatal care (ANC) |  |
| Two-month long media campaign targeted at adolescents |  |
| Sex and reproductive education at schools |  |
| Mobile messaging for pregnant women |  |
| ***2: Prevention and Screening*** | **3** |
| Cervical cancer screening (three per lifetime) |  |
| HPV vaccine at schools |  |
| Contraceptive provision at schools |  |
| Improve and provide more ANC- training of healthcare workers in basic ANC |  |
| Exclusive breastfeeding – promotion and access to lactation specialists |  |
| Complementary feeding- demonstrations |  |
| ***3: Treatment*** | **2** |
| Expanded services for termination of pregnancy - make available in communities. |  |
| Dedicated obstetric ambulances |  |
| Maternity waiting homes |  |
| Labour and delivery management |  |
| emergency care for mothers and newborn |  |
|  |  |
| **Child health** |  |
| ***1: Education and information*** | **1** |
| media campaigns for immunisation and handwashing |  |
| workshops on child health |  |
| ***2: Prevention*** | **1** |
| Hand washing promotion in community |  |
| Provision of food supplements for malnutrition & education |  |
| Immunisations (at primary health care level) |  |
| ***3: Treatment*** | **1** |
| Oral rehydration solution for diarrhoea |  |
| Oral antibiotics : case management of pneumonia in children |  |
|  |  |
| **HIV/AIDS &TB** |  |
| ***1: Education and information*** | **1** |
| 2 months long media campaign |  |
| 1 education workshop per year in every secondary school |  |
| ***2: Prevention and Screening*** | **5** |
| Increase provision of condoms |  |
| Youth friendly medical-male-circumcision services - include school friendly hours |  |
| testing for HIV exposed babies |  |
| HIV Counselling and Testing |  |
| Making HIV Counselling and Testing youth friendly (training; extra hours) |  |
| ***3: Treatment*** | **11** |
| ARVs & mobile messaging reminders for adherence |  |
| Prevention of mother to child transmission of HIV (ARVs and breastfeeding choices) |  |
| TB treatment |  |
| Home based care |  |
| STI treatment |  |
| Youth Care Club |  |
|  |  |
| **Lifestyle diseases/ non-communicable diseases^[[1]](#footnote-1)^ (diabetes, hypertension, cancer)** |  |
| ***1: Education and information*** | **1** |
| Two-month long media campaign on lifestyle diseases |  |
| Educational workshop on lifestyle diseases at community level |  |
|  |  |
| ***2: Prevention and screening*** | **1** |
| School vegetable garden |  |
| Increase screening and counselling in communities |  |
| ***3: chronic medication*** | **17** |
| Diabetic medication |  |
| hypertension medication |  |
| Mobile messaging for adherence |  |
| ***4. Treatment for complications and rehabilitation*** | **6** |
| Retinopathy |  |
| Dialysis |  |
| Amputations |  |
| Chemotherapy and radiation |  |
| Rehab session for stroke patients |  |
| ***5: Palliative care*** | **1** |
| Palliative care (in-patient) |  |
| Palliative home based care |  |
|  |  |
| **Access** |  |
| 1: Improve staff attitudes (especially around family planning services for adolescents)  and improve management and monitoring and evaluation in clinics | 1 |
| 2: Make clinics operational for longer hours | 4 |
| 3: Increase number of mobile clinics from 5 to 10 | 1 |
| 4: Chronic Medicines (ARVs, diabetes meds, hypertension meds) available at community health centres | 2 |
| 5: Increase number of nurses in clinics  and more pharmacists in clinics to dispense meds | 1 |
|  |  |
| **Woman and Child abuse** |  |
| ***1: Education and information*** | **1** |
| Education/ life skills for children and adolescents, workshops on gender |  |
| Media messaging |  |
| Training and support workshops for families |  |
| ***2: Management of rape and abuse*** | **1** |
| Care and support programmes, including counselling and comfort kit |  |
| Training of nurses |  |
| ***3: Treatment*** | **1** |
| Treatment of injuries at clinics |  |
| Post-exposure prophylaxis 4 weeks |  |
|  |  |
| **Malaria** |  |
| ***1: Education and information*** | **1** |
| Annual education campaign |  |
| ***2: prevention and screening*** | **1** |
| ITN & indoor residual spraying |  |
| screening at clinics |  |
| ***3: Treatment*** | **1** |
| antimalarial medication for uncomplicated cases |  |

*Holes represent cost of the intervention with one hole equivalent to approximately 1.5% of the total cost of the package

1. Lifestyle diseases as a term was preferred by the community over non-communicable diseases (NCDs) during modification of the CHAT tool. Lifestyle diseases was retained in the CHAT board and user manual but NCDs is used throughout the paper to refer to these conditions. [↑](#footnote-ref-1)
