# Supplementary material for: Moving towards social inclusion: Engaging rural voices in priority setting for health
Source: Health Expect. 2023 Oct 26;27(1):e13895. doi: 10.1111/hex.13895 (PMC10726206; doi:10.1111/hex.13895)
Supplement: Supplementary file 2 — Supporting information. [file HEX-27-e13895-s003.docx]

**Appendix C:** CHAT process

**Pre-exercise:** Participants complete a short, self-administered questionnaire

**Introduction:** Facilitator explains the CHAT board and user manual

**Round 1: Individual priorities**

Participants individually allocate 35 stickers to health issues and interventions for their own family using individual CHAT boards and user manuals

**Round 2: Group discussion and prioritisation**

- Group allocates 35 stickers on a single CHAT board for community priorities
- Facilitator uses scenario cards to guide thinking, and refers to the user manual for detail of interventions
- Participants discuss topics in depth and deliberate
- Agreement reached through majority vote for group sticker allocations

**Round 3: Individual priorities**

Participants individually allocate 35 stickers to health issues and interventions for their own family again

**Conclusion:** Facilitator asks some brief feedback questions to the group and concludes the exercise
