# Supplementary material for: Moving towards social inclusion: Engaging rural voices in priority setting for health
Source: Health Expect. 2023 Oct 26;27(1):e13895. doi: 10.1111/hex.13895 (PMC10726206; doi:10.1111/hex.13895)
Supplement: Supplementary file 3 — Supporting information. [file HEX-27-e13895-s001.docx]

**Appendix A:** CHAT SA tool as adapted for Rural Bushbuckridge South Africa


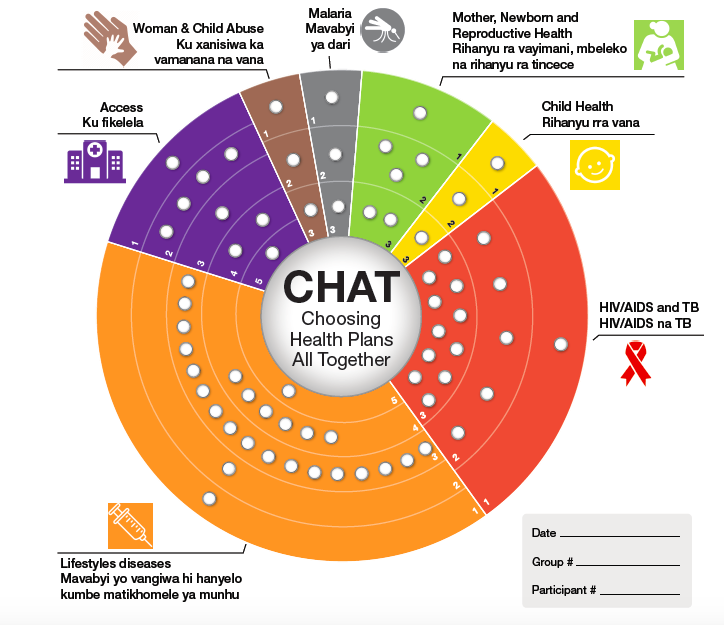


originally published in Tugendhaft, A., Danis, M., Christofides, N., Kahn, K., Erzse, A., Gold, M., Twine, R., Khosa, A., Hofman, K. (2020). 'CHAT SA: Modification of a Public Engagement Tool for Priority Setting for a South African Rural Context', *International Journal of Health Policy and Management*, (), pp. -. doi: 10.34172/ijhpm.2020.110

| **Key** |  |
| --- | --- |
| 1: Education and information | **Access** |
| 2: Prevention and screening | 1. Improve staff attitudes |
| 3: Treatment | 2. Clinics operational for longer hours |
| 4. Treatment for complications and rehab | 3. Increase mobile clinics |
| 5: Palliative care | 4. Chronic meds available at community health centres |
|  | 5. Increase number of nurses and pharmacists in clinics |
